# Empower your child's health: tailored strategies to prevent rhinosinusitis

**DOI:** 10.1016/j.jped.2024.02.005

**Published:** 2025-06-18

**Authors:** Francesca De Bernardi, Chiara Zeroli, Sandra Coecke, Massimo Landi, Stefania Gallo, Paolo Castelnuovo, Luana Nosetti

**Affiliations:** a Division of Otorhinolaryngology, ASST Sette Laghi, Varese, Italy; b Division of Otorhinolaryngology, ASST della Valle Olona, Busto Arsizio, Italy; c European Commission, Joint Research Centre (JRC), Ispra, Italy; d Department of Medical Sciences, graduate School of Allergology and Clinical Immunology, University of Turin, Turin, Italy; e Department of Biotechnology and Life Sciences, University of Insubria, Varese, Italy; f Department of Medicine and Surgery, Pediatric Unit, University of Insubria, "F. Del Ponte" Hospital, Varese, Italy

**Keywords:** Prevention, Rhinosinusitis, Sinusitis, Upper respiratory tract infections, Mental health, Depression

## Abstract

**Objective:**

This review explores preventive strategies for pediatric rhinosinusitis and examines their potential impact on children's mental well-being, advocating for a comprehensive holistic approach that includes medical disciplines and government policies to support EU and global prevention strategies.

**Sources:**

A comprehensive search encompassed Medline, Embase, PubMed, and the Cochrane Library for English-language articles from January 2010 to December 2023. Inclusion criteria involved papers on pediatric rhinosinusitis prevention in the pediatric population, published in peer-reviewed journals. Following the removal of duplicates and exclusion of irrelevant studies, 20 unique titles were included in the review.

**Summary of the findings:**

The review underscores the challenges posed by the similarity of symptoms between pediatric rhinosinusitis and other common childhood illnesses. It emphasizes the interconnected nature of upper and lower airways, illustrating the potential impact on both physical and mental well-being in children. The findings highlight the necessity for a multifaceted prevention approach, supported by individualized prevention plans, medical professional involvement, and government policies.

**Conclusions:**

The holistic research and clinical approach proposed in this review contribute valuable insights into the global efforts aimed at reducing the incidence of pediatric rhinosinusitis while promoting the mental well-being of children. The article serves as an informative resource for readers seeking a deeper understanding of pediatric rhinosinusitis prevention strategies.

The review emphasizes the necessity of a multidisciplinary approach, involving pediatricians, otolaryngologists, pneumologists, and allergologists, in PRS prevention. Government prioritization of preventive measures is essential. Precision medicine and integrative approaches are recommended for tailored treatment plans.

## Introduction

**Figure 1:**
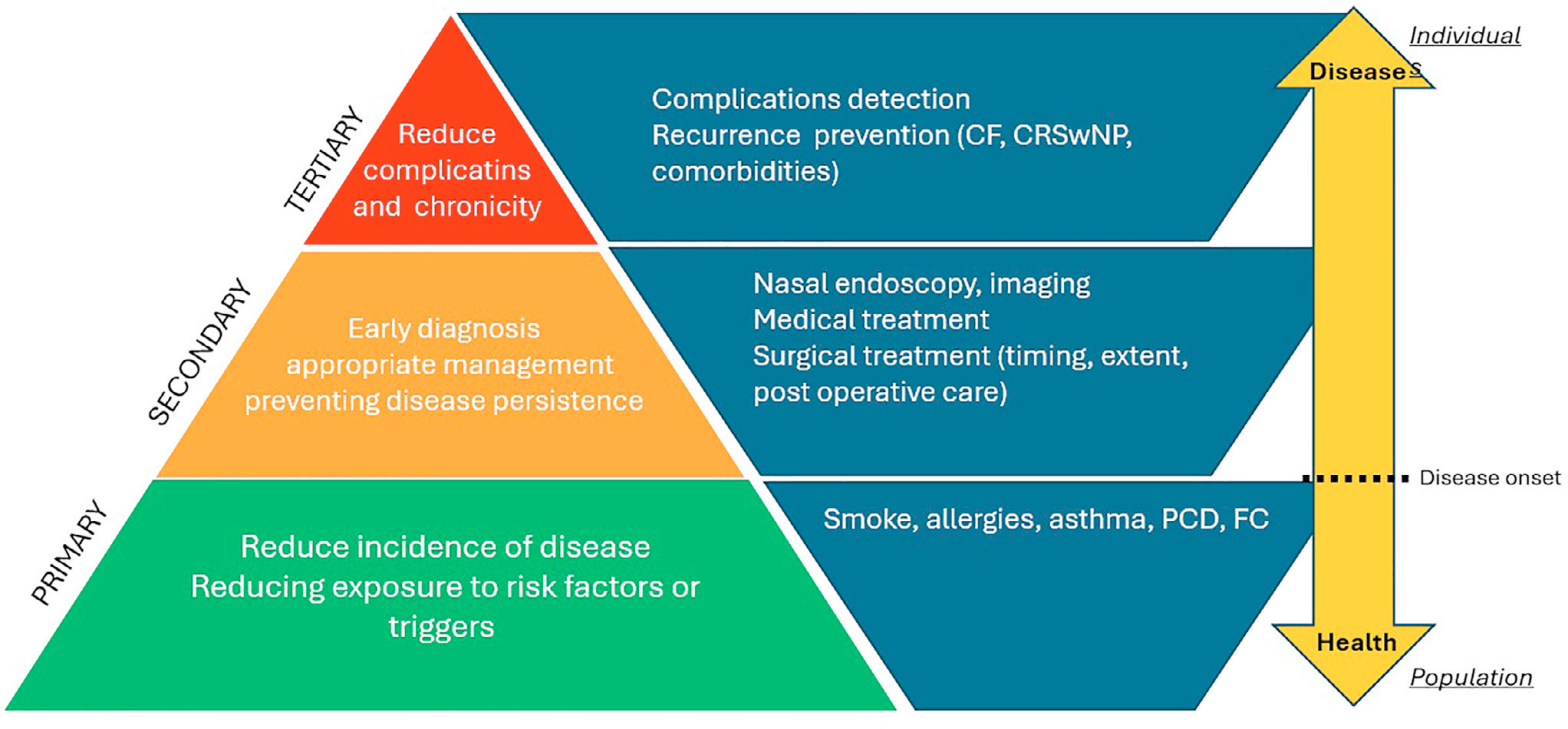
Pediatric rhinosinusitis prevention divided into primary, secondary and tertiary.

The relevance of prevention is highly emphasized in current times, as public health prioritizes disease prevention and health promotion over mere diagnosis and treatment of illnesses. In December 2021, the European Commission launched the Healthier Together – European Union (EU) non- communicable diseases (NCD) initiative to support EU countries in identifying and implementing effective policies and actions to reduce the burden of major NCDs, improve citizens’ health and well- being and promote a holistic and coordinated approach to prevention and care. In June 2022, the EU launched an initiative on mental health with the goal of ensuring that all EU citizens have access to prevention, early diagnosis, and high-quality healthcare.^1^^,^^2^ The word prevention is derived from the Latin “prae-venire,” meaning “to come first.” Prevention starts before the onset of a disease, aiming to early identify and provide appropriate treatment, thereby preventing its progression into chronicity or complications (Figure 1). The COVID-19 pandemic has highlighted the significance of preventing highly contagious communicable diseases, but prevention is also crucial in less common but more severe NCDs, with varying strategies for implementation.

Pediatric rhinosinusitis (PRS) is a common condition whose true epidemiology is not well described yet, but it is estimated around 14.6 % worldwide.^3^ However, the prevalence is higher in certain patient groups such as those affected by allergies or immunosuppression. It can be frequent and recurrent causing discomfort and deterioration in quality of life (QoL) or can be particularly alarming due to potential complications such as the spreading of infection to the eyes or brain. Besides, the concept of United Airway Disease suggests that diseases of the upper and lower airways are linked and should not be treated as separate entities. While the mechanisms behind this link are not fully explained, evidence supports the idea that prevention measures taken in one area may also benefit the other.^4^^,^^5^

An effective strategy to prevent PRS involves a comprehensive approach that is tailored to the specific needs and age of each child. Such an approach can enhance children QoL and help prevent potentially severe complications. A multidisciplinary approach is crucial for the prevention of PRS, involving specialized medical professionals like pediatricians, otolaryngologists, pneumologists, and allergologists, who have the knowledge and skills to diagnose and treat this condition. With their expertise and specialized tests, they can determine the underlying causes of PRS and develop appropriate treatment plans, while also providing guidance on preventive measures to parents and caregivers. However, it is also important for governments to prioritize preventative measures in healthcare policies to help reduce the incidence of illnesses and improve public health overall.

## Methods

### Search strategy

The authors investigated the association between prevention, rhinosinusitis, and children by conducting comprehensive research on high-quality evidence sources, including Medline, Embase, PubMed, and Cochrane Library. The exact search terms used were "prevention," "sinusitis," and "children." The research was restricted to human studies published in the English language from 2010 to the present, and all relevant publications were considered.

The relevant papers were extracted independently by two authors (FDB and CZ) for inclusion in this review, which was then reviewed and revised by all authors. A limitation of the study is the restriction of the review to articles published only in the English language.

### Inclusion and exclusion criteria

The included papers needed to address a specific research question related to the prevention of rhinosinusitis or strategies for preventing rhinosinusitis in the pediatric population. Additionally, they had to be published in peer-reviewed journals or reliable technical reports.

The exclusion criteria included the absence of a specific research question on rhinosinusitis prevention, non-English publications, studies in the adult population, absence of reported outcomes on preventing rhinosinusitis, case reports and case series. All included studies were classified into primary, secondary, and tertiary prevention categories.

## Results

**Figure 2:**
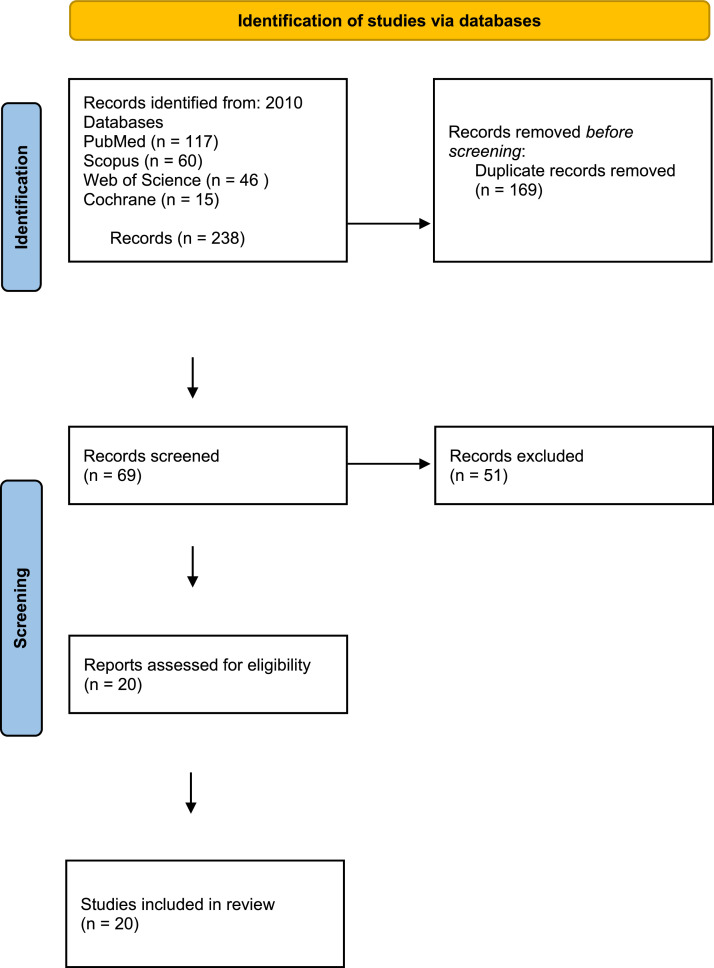
Study search, selection, and inclusion process.

The initial search yielded 238 publications from 2010 to 2023, which were reduced to 169 after removing duplicates. Once irrelevant studies were excluded, the remaining 20 unique titles were independently screened and assessed for eligibility by the authors. This review summarizes the findings of these 20 papers, which are categorized based on different types of prevention (Figure 2). Included articles and principal results are listed in Table 1.^6^, ^7^, ^8^, ^9^, ^10^, ^11^, ^12^, ^13^, ^14^, ^15^, ^16^, ^17^, ^18^, ^19^, ^20^, ^21^, ^22^, ^23^

**Table 1:** Articles included in the qualitative analysis and reviewed

Primary Prevention		
First Author (year)	Type of Study (n. of patients)	Results
Veskitkul J. (2017)^6^	Randomized, double-blind, placebo-controlled study (40 children,5–15 years)	- Azithromycin prophylaxis can reduce the number of rhinosinusitis episodes and medication score and improve nasal symptoms in nonallergic rhinitis children with recurrent acute rhinosinusitis.
Bellussi L.M. (2019)^7^	Review	- The existing microbial ecosystem have a central role in the pathogenesis of respiratory disease; - New therapeutic approaches include the implantation and persistence within the normal microflora of relatively innocuous "effector" bacteria that can competitively exclude or prevent the outgrowth of potentially disease-causing bacteria; - Bacteriotherapy in children have beneficial effects in the prevention of upper respiratory tract infections.
Chen J. (2017)^8^	Randomized control study (96 children, 4–12 years)	- Bacterial lysate used in the remission period of rhinosinusitis in children was shown to provide long-term prophylaxis; - Bacterial lysate can reduce the frequency of rhinosinusitis attacks and ameliorate attack symptoms.
Cingi C. (2015)^9^	Review	- Antileukotrienes are a promising treatment for upper airway diseases as evidenced by a detailed literature review. Specifically, montelukast treatment following sinus surgery has been found to significantly reduce eosinophilic cationic protein levels in the bloodstream and improve nasal and pulmonary symptoms and antileukotrienes have been shown to effectively treat allergic rhinitis
Ciprandi G. (2020)^10^	Review	- Local Bacteriotherapy significantly reduced the number of respiratory infections, their severity, the use of antibiotics, and school absences; - Local Bacteriotherapy is a promising approach in preventing respiratory infections and could be a routine strategy to contrast infections in the future.
Chiappini E. (2021)^11^	Consensus document	- The panel agrees that it is necessary to assesses and contains environmental risk factors, to support the administration of vaccines and to improve the best practices to reduce respiratory infections, such as hand washing.
Passali D. (2019)^12^	Prospective observational study (202 children, median age 7,5 years)	- Prophylactic bacteriotherapy by administration of Streptococcus salivarius 24SMB and Streptococcus oralis 89a in children with a history of recurrent upper respiratory tract infection could reduce the number of episodes of otolaryngologic infections. - Bacteriotherapy can be even more clinically important due to increasing difficulty in finding new effective antibiotic compounds. - New alternative therapeutic approaches must be found with, in comparison to antibiotics, greater specificity and safety with respect to patients' native beneficial flora; lack of drug interactions; the ability to leverage complementary systemic modes of action; and drastically reduced risk of developing resistance within the patient population and the environment.
Piqué N. (2018)^13^	Review	- In clinical trials, xyloglucan has been seen to reduce symptoms of nasal disorders. - Xyloglucan, endowed with film-forming protective barrier properties, is a safe non-pharmacological alternative for the management of different diseases, such as gastrointestinal and nasal disorders.
Principi N. (2017)^14^	Review	- Methods for performing nasal irrigation have to be standardized to determine which solution, device, and duration of treatment are adequate to obtain favorable results. This seems particularly important for children that suffer from a great number of sinonasal problems and might benefit significantly from an inexpensive and simple preventive and therapeutic measure such as NI.
Tarantino V. (2019)^15^	Prospective observational study (80 children, mean age 5.26±2.52 years)	- Bacteriotherapy using Streptococcus salivarius 24SMB and Streptococcus oralis89a nasal spray could prevent recurrent UI in children.
Fokkens W.J. (2020)^16^	Guidelines	- Occupational and environmental factors are of increasing importance in primary prevention and the effects of global warming carefully monitored. - Co-morbidities such as allergy, asthma and GORD should be considered. - Genetic and microbiological factors will likely become of greater importance.

Secondary Prevention		
First Author (year)	Type of Study	Results
Fokkens W.J. (2020)^16^	Guidelines	- Early diagnosis and selection of the optimal treatment is central in URTI secondary prevention; - Optimizing medical treatment and consideration of the timing and extent of surgery can improve outcomes.
Kucuksezer U.C. (2018)^17^	Review	- The exploration of disease endotypes and introduction of novel agents are important advancements in underlying CRS pathogenesis and disease-contributing factors. - Prior studies performed without disease-endotyping resulted in the inefficiency of certain drugs and insignificant results. - The identification of biomarkers, development of personalized approaches, and utilization of disease algorithms are required for CRS therapy success.
Hamilos DL (2015)^18^	Review	- Differences in the microbiome could be a cause of the substantial differences in the symptoms of and treatment options for adult and pediatric patients with chronic rhinosinusitis and using microbiome analysis to understand the pathogenesis of CRS in adults and children is crucial; - Actinobacteria had a significantly higher relative abundance in the adult group than in the pediatric group at the phylum level. - At the genus level, Corynebacterium showed significantly higher relative abundance in the adult group than in the pediatric group.
Abzug M.J. (2014)^19^	Review	- Antibiotics probably do have a role in the treatment of pediatric ABS - The most compelling rationale is prevention of serious complications, but proof for this rationale is lacking.
Babar-Craig H. (2010)^20^	Review	- Complications which require surgical treatment are similar in both a “prior antibiotic treated group” and a “no prior antibiotic group”, suggesting limited benefit of oral antibiotics in the Primary Care setting.

Tertiary Prevention		
First Author (year)	Type of Study	Results
Cantone E. (2022)^21^	Systematic Review	- In children aged over 9 years, large subperiosteal orbital abscesses,impaired vision, ophthalmoplegia, proptosis, elevated C-reactive protein and absolute neutrophil counts, hemodynamic compromise, no clinical improvement after 48/72 h of antibiotic therapy, and a Chandler III score or higher are clinical markers of the need for surgery (criticalities: most of the studies are observational and retrospective, and further studies are needed to identify reliable and repeatable clinical markers of the need for surgery).
Fokkens W.J. (2020)^16^	Guidelines	- In tertiary prevention, a careful review of ongoing treatment, technique and compliance with medication should be undertaken. - Growth in digital healthcare and patient apps may encourage self-management and increase compliance.
Rohde R.L. (2022)^22^	Review	- Determination of the optimal approach depends on patient clinical features including age, history of prior endoscopic sinus surgery, and presence of intracranial involvement on presentation. - An individualized, targeted, and interdisciplinary approach to the treatment of Pott's Poffy Tumor is critical for successful disease resolution
Hansen F.S. (2012)^23^	Retrospective cohort study (47 patients of which 26 children, total mean age 35.9 years)	- Antibiotic treatment of acute rhinosinusitis in general practice does not play a role in preventing complications.

## Discussion

### Reduce exposure to risk factors (primary prevention)

Primary prevention reduces the incidence of PRS by reducing exposure to risk factors or triggers. PRS is initiated by both environmental factors and genetic predisposition, which lead to inflammatory changes, mucociliary dysfunction, and alterations in the microbial community. The exposome is a measure of all the exposures an individual experiences throughout their lifetime and how those exposures relate to health. An individual's exposure begins before birth and includes influences from environmental and lifestyle factors that can have long-term effects on mental health.^24^^,^^25^

Possible strategies that have been investigated to decrease the occurrence of PRS include encouraging a healthy lifestyle, enhancing the body's immunity, avoiding harmful behaviors, and establishing an environment with minimal exposure to toxins.^26^ Environmental factors such as air pollution, cigarette smoke, occupational exposures, and allergens can all contribute to rhinosinusitis by triggering or exacerbating inflammation.

By better understanding the exposome of individuals with rhinosinusitis, researchers may be able to identify specific environmental factors that contribute to the development of the disease and develop targeted prevention and treatment strategies. Air pollution, especially ozone and particulate air pollutants like diesel exhaust particles, can trigger asthma symptoms by causing inflammation in the airways through specific inflammatory pathways.^27^

Tobacco smoke can impair mucociliary clearance and is a potential exacerbating factor for chronic rhinosinusitis (CRS). In vitro studies show that cigarette smoke extract can impair ciliogenesis in a dose-dependent manner.^28^ Passive smoking in childhood is associated with CRS. Exposure to second-hand smoke and active smoking may also alter the normal bacterial flora in the nasopharynx, but this effect can be reversed following smoking cessation. Smoking prevention and cessation efforts may be effective in reducing the risk of rhinosinusitis in individuals who smoke or are exposed to second-hand smoke.^28^, ^29^, ^30^, ^31^

Personal characteristics, such as genetics and allergy sensitization, can also play a role in predisposing individuals to rhinosinusitis. Some genetic disorders like cystic fibrosis and primary ciliary dyskinesia are associated with a higher prevalence of CRS, but they are only a small proportion.^32^ Allergies, particularly allergic rhinitis, can also be a predisposing factor for sinusitis. Mucosal edema within the ostiomeatal complex in allergic rhinitis may obstruct sinus ostia, leading to mucus retention and infection.^33^

Adenoids, as part of the immune system, may contribute to the onset of rhinosinusitis in children. When adenoids are enlarged, due to repeated infections, they can block the drainage pathways of the mucus, which can lead to sinusitis. Children with enlarged adenoids may experience symptoms such as nasal congestion, runny nose, postnasal drip, cough, and fever. Nasal endoscopy can help to diagnose the underlying cause of symptoms, and if the sinusitis is caused by enlarged adenoids and sinusitis episodes are recurrent and disabling, the otolaryngologist may decide together with the caregiver not to wait for their normal involution with growth but to surgically intervene by reducing their size.^16^^,^^34^

Most papers included in the primary prevention appear to focus mainly on medical therapy for preventing rhinosinusitis, rather than on personal or environmental factors that may predispose individuals to develop the condition. One article suggests that azithromycin prophylaxis can reduce the number of rhinosinusitis, but due to limited efficacy and high risk of resistance, it is not recommended.^6^

A promising new approach involves introducing bacteria into the microflora to prevent the growth of harmful ones. Studies on bacteriotherapy in children with upper respiratory tract infections have shown it to be safe and effective, making it a viable therapeutic strategy for consideration. Further research is needed to fully understand the potential benefits of this approach.^7^^,^^10^

The use of bacterial lysate in children during the remission period of rhinosinusitis has been found to provide long-term prophylaxis. This approach is effective in reducing the frequency of rhinosinusitis exacerbations and improving symptoms during attacks.^8^

Antileukotrienes have demonstrated the ability to decrease levels of eosinophilic cationic protein in the bloodstream and improve both nasal and pulmonary symptoms. They have been proven effective in treating allergic rhinitis, but there should be monitoring for possible adverse psychiatric effects.^35^ However, the EPOS2020 steering group does not recommend the addition of montelukast to nasal corticosteroids for adults and does not endorse the use of anti leukotrienes in children.^9^^,^^16^

### Early diagnosis and appropriate treatment (secondary prevention)

Secondary prevention aims to detect and intervene in diseases at their earliest stages, targeting healthy-appearing individuals with subclinical forms of the disease. Its emphasis is on early disease detection and achieving disease and symptom control through optimal treatment.^36^ Recently updated European guidelines on rhinosinusitis and nasal polyps reflect the importance of secondary prevention.^16^

### Early diagnosis

Nasal congestion and discharge are common symptoms in children and may indicate the presence of sinusitis, but they are not specific enough to make an accurate diagnosis on their own. Therefore, relying solely on symptoms to define sinusitis may lead to an overestimation of its prevalence.^37^ Children often complain of obstructive symptoms, but the underlying causes can vary, such as adenoid hypertrophy, hypertrophy of the inferior turbinates, or nasal septum deviation. Children with rhinosinusitis may have a persistent cough, particularly at night.

If a child is experiencing any of these symptoms, it is important to perform a physical examination observing the breathing pattern during respiration (open mouth), anterior rhinoscopy (nasal crusting, nasal septum deviation, varices, and inferior turbinate hypertrophy), otoscopy evaluating for middle ear effusion, assessment for tonsillar hypertrophy, palpating adenopathy at the neck and nasal endoscopy.^38^ An endoscopic examination is an effective method to identify the cause of the problem and to ensure that appropriate treatment is started as soon as possible. When performing nasal endoscopy, it is possible to collect purulent discharge to obtain an antibiogram and set up targeted antibiotic therapy.

All these signs and symptoms can lead to the diagnosis of rhinosinusitis. If the condition persists for more than 12 weeks, it is termed CRS.^16^

Current guidelines recommend the use of CT for diagnosing pediatric CRS and recent advancements in pediatric CT scan (cone bean) have made it possible to minimize radiation exposure without compromising the quality of imaging. In cases of complicated RS that require assessment for intracranial or orbital involvement, MRI is the recommended imaging modality. Both the AAO—HNS and EPOS advise against the use of plain radiographs.^39^ Serum-specific immunoglobulin E (IgE) testing and skin prick testing can be considered in children with features of allergic rhinitis to reach the diagnosis.

### Optimal treatment paradigm

Antibiotics are believed to play a role in treating pediatric acute bacterial rhinosinusitis. The guidelines from the American Academy of Pediatrics recommend using amoxicillin with or without clavulanate as the initial therapy. However, a study of commercially insured children with acute sinusitis revealed that a significant number of patients were prescribed non-first-line or multiple antibiotics. Given that antibiotics are ineffective for the common cold or persisting acute purulent rhinitis and can lead to adverse effects, interventions are necessary to promote appropriate antibiotic use, especially in light of the global crisis of antibiotic resistance.^16^^,^^40^

According to the EPOS guidelines, initial treatment for patients with CRS typically involves medical therapy. However, if medical treatment proves unsuccessful, surgical intervention may be necessary. Adenoidectomy is often recommended as the safest and most straightforward surgical option, with functional endoscopic sinus surgery (FESS) considered as a later option.^16^

### Prevent complications and reduce relapses (tertiary prevention)

#### Preventing complications

The complications of ABRS are uncommon (estimated at 1 in 12,000 cases) but potentially life-threatening events. Table 2 lists symptoms of alarm that should prompt early medical attention. There seems to be no significant difference in the frequency of severe complications between countries with low antibiotic usage and those with high rates of antibiotic prescription.^23^

**Table 2:** Alarm symptoms (modified EPOS)

Symptoms	Pathology/(Frequency)	Subtypes
Periorbital oedema/erythema Displaced globe Double vision Ophthalmoplegia Reduced visual acuity	Orbital complication/(60–80 %)	preseptal cellulitis, orbital cellulitis, subperiosteal, intraorbital abscess
Severe headache Signs of meningitis Neurological signs Ophthalmoplegia	Endocranial complication/(15–20 %)	epidural empyema, subdural empyema, brain abscess, meningitis, encephalitis, superior sagittal and cavernous sinus thrombosis.
Frontal swelling	Osseous complications/(5 %)	osteomyelitis and may present as a subperiosteal frontal bone abscess (Potts Puffy tumour) or a frontocutaneous fistula.
Unilateral symptoms Bleeding Crusting Cacosmia	Neoplasm	

It is crucial to promptly diagnose and treat complicated sinusitis with intravenous antibiotics and/or surgical drainage to avoid long-term complications and mortality. Imaging, particularly CT and MRI, is necessary to determine the extent of involvement of adjacent structures. Consultation with an ophthalmologist is recommended for objective assessment of proptosis, orbital pressure, visual acuity, color vision and eye movements, which should be thoroughly documented for clinical and medicolegal purposes.^41^

Orbital cellulitis and periorbital cellulitis are the most prevalent complications in children. Medical management is typically recommended, with surgery reserved for severe cases, but there is a lack of agreement on the clinical indicators that would necessitate surgical intervention. A systematic review suggests that children with large subperiosteal orbital abscesses, impaired vision, ophthalmoplegia, proptosis, elevated C-reactive protein and absolute neutrophil counts, hemodynamic instability, a Chandler III score or higher, and no clinical improvement after 48/72 h of antibiotic therapy may require surgery.^21^

### Manage long-term follow up and improve quality of life

Children with rhinosinusitis have been found to experience major pain and discomfort, limitations in social functioning, and decreased quality of life compared to peers without the condition.^42^ Symptoms of allergic rhinitis, such as nasal obstruction and rhinorrhea, which are also reported in patients with sinusitis, can be distracting for children at school, potentially leading to decreased academic performance. Sleep disorders seen in allergic rhinitis, primarily caused by nasal congestion and obstruction, tend to be at their worst during the night when a child is lying down to sleep.^35^^,^^43^ The comorbidity of depression with CRS is a significant concern, with higher rates compared to the general population.^44^^,^^45^ Patients with CRS and comorbid depression experience a poorer quality of life and potentially worse treatment outcomes.^46^ This issue is also apparent in the adolescent population, highlighting the importance for healthcare professionals to routinely screen and assess for depression in children with CRS and integrate mental health support into their care.^47^^,^^48^ Early identification, appropriate interventions, and a holistic approach can improve outcomes and enhance the overall well-being of children with CRS and comorbid depression.

Untreated sinusitis can also have a negative impact on mental health. Educating patients and their families about the condition and its treatment can help them better understand the impact of sinusitis on their quality of life, potentially reducing anxiety and stress related to the condition. Regular assessments of the patient's quality of life can help identify any changes or declines associated with sinusitis, allowing clinicians to intervene early and prevent further mental health complications. Referral to mental health professionals may be necessary if the patient is experiencing significant mental health issues related to sinusitis.^42^

To manage long-term follow-up and improve the quality of life, it is important to take a comprehensive approach to care that addresses both the physical and mental health outcomes of children with rhinosinusitis.

### Directions for future research

To implement effective strategies for preventing rhinosinusitis, engaging a multidisciplinary healthcare team is essential. The diverse expertise of various specialists allows for a more thorough identification and understanding of the issue. Therefore, the recommendation is to conduct a multidisciplinary visit where multiple specialists can simultaneously attend to the patient. This collaborative approach enhances the comprehensive assessment and management of rhinosinusitis, leveraging the diverse skills of healthcare professionals. Specialists must adhere to the guidelines for rhinosinusitis, as this allows for improving the accuracy of diagnoses and the effectiveness of treatments. Additionally, it is possible to develop standard in-hospital protocols for identifying the risk factors of sinusitis and addressing them promptly. This systematic approach allows for the proactive identification and management of factors that could contribute to the development of rhinosinusitis in children.

The creation of a pediatric rhinosinusitis patient registry with multicenter collaborations could also enable a better understanding of the epidemiology and identification of potential risk factors for the disease. This registry facilitates information sharing among healthcare facilities, promoting research and the implementation of evidence-based preventive strategies.

In addressing rhinosinusitis, it is also crucial to integrate mental health screenings and interventions as part of routine care for pediatric rhinosinusitis patients, aiming to identify and address mental health issues promptly and provide the necessary support and resources. This holistic approach recognizes the importance of mental health in the overall management of pediatric rhinosinusitis.

A standardization process ensures consistent and optimized practices, intending to enhance the overall quality of care provided to pediatric patients with rhinosinusitis.

## Conclusion

EU and global efforts to reduce the incidence of communicable and non-communicable respiratory pathologies have highlighted the importance of innovative preventive approaches. Therefore, it is crucial to involve all stakeholders and identify comprehensive measures for the prevention and implementation of a holistic and coordinated approach to the prevention and care of rhinosinusitis. Children are particularly vulnerable to upper respiratory infections and rhinosinusitis, and there is also increasing data on children's overall well-being, including mental health and depression. This highlights the importance of combining precision medicine and integrative approaches to identify the underlying causes of nasal obstruction and develop tailored treatment plans that address both physical and mental well-being.

This paper aims to outline strategies for preventing sinusitis in children, including the identification of high-risk children, early diagnosis through identifying affected individuals from a larger group with nasal obstruction, and prompt recognition and treatment of complications. Precise diagnosis is crucial to avoid diagnosing rhinosinusitis only when complications have already developed. The paper also discusses the use of new technologies in diagnosis and treatment, emphasizing the importance of addressing an often underestimated respiratory pathology in children.

## Conflicts of interest

The authors declare no conflicts of interest.
